# Biopsy of the facial nerve in slow-onset facial palsy

**DOI:** 10.1007/s00405-024-08826-3

**Published:** 2024-07-13

**Authors:** Gianluca Sistori, Michael Götting, Cornelia Radke, Markus Jungehülsing

**Affiliations:** 1 Klinik für Hals-, Nasen- und Ohrenheilkunde, Ernst von Bergmann Klinikum Potsdam, Charlottenstraße 72, 14467 Potsdam, Germany; 2 Institut für Pathologie, Ernst von Bergmann Klinikum Potsdam, Charlottenstraße 72, 14467 Potsdam, Germany

**Keywords:** Case report, Malignant neoplasia of the parotid gland, Primary squamous cell carcinoma, Slow-onset peripheral facial palsy, Perineural tumor spread

## Abstract

**Introduction:**

Primary squamous cell carcinoma of the parotid gland typically presents as a palpable, often painless mass. Peripheral facial palsy as the only sign of malignant neoplasia is rare. In these cases, the diagnosis is regularly confirmed by radiological imaging followed by surgical exploration and biopsy. However, if there is no detection of malignant lesions and no evidence of a tumor, the reluctance to take a biopsy of an unremarkable nerve can lead to misdiagnoses.

**Case report:**

A 40-year-old female patient without medical history presented to our clinic with a complete right-sided peripheral facial palsy that had slowly progressed for 2.5 years. All other otorhinolaryngological examination findings were within normal limits. Magnetic resonance imaging examination of the head and neck and 18-fluorodeoxyglucose positron emission tomography showed unremarkable results. We proceeded with surgical exploration, which revealed no evidence of a tumor and an externally completely unremarkable facial nerve. A biopsy from the main trunk area of the nerve revealed an infiltration by a squamous cell carcinoma. Total parotidectomy with resection and reconstruction of the facial nerve and neck dissection was performed. Considering the absence of a primary tumor and other tumor formations the diagnosis of a completely regressive primary squamous cell carcinoma of the parotid gland was confirmed.

**Conclusion:**

In conclusion, in the case of slow-onset peripheral facial palsy that persists without signs of recovery, a gadolinium-enhanced MRI should be performed. If imaging is unremarkable and there is no primary tumor detection along the course of the facial nerve, a surgical exploration with biopsy of the facial nerve is necessary.

## Introduction

Malignant neoplasms of the salivary glands can be classified into a variety of subtypes. Primary squamous cell carcinoma of the parotid gland represents a rare yet highly aggressive histological subtype that is often associated with a poor prognosis. Clinically, it typically presents as a palpable and often painless mass. Facial and ear pain, as well as peripheral facial palsy due to neuronal invasion and perineural spread of the malignant cells, usually develop in advanced stages [1].

Cases of slow-onset peripheral facial palsy as the only sign of malignant neoplasia of the parotid gland are rare, and can be mistaken for idiopathic facial palsy, such as Bell’s palsy [2]. Therefore, slow-onset peripheral facial palsy without signs of regeneration should be considered suspicious for malignancy until proven otherwise and requires further diagnostics.

## Case report

**Fig. 1:**
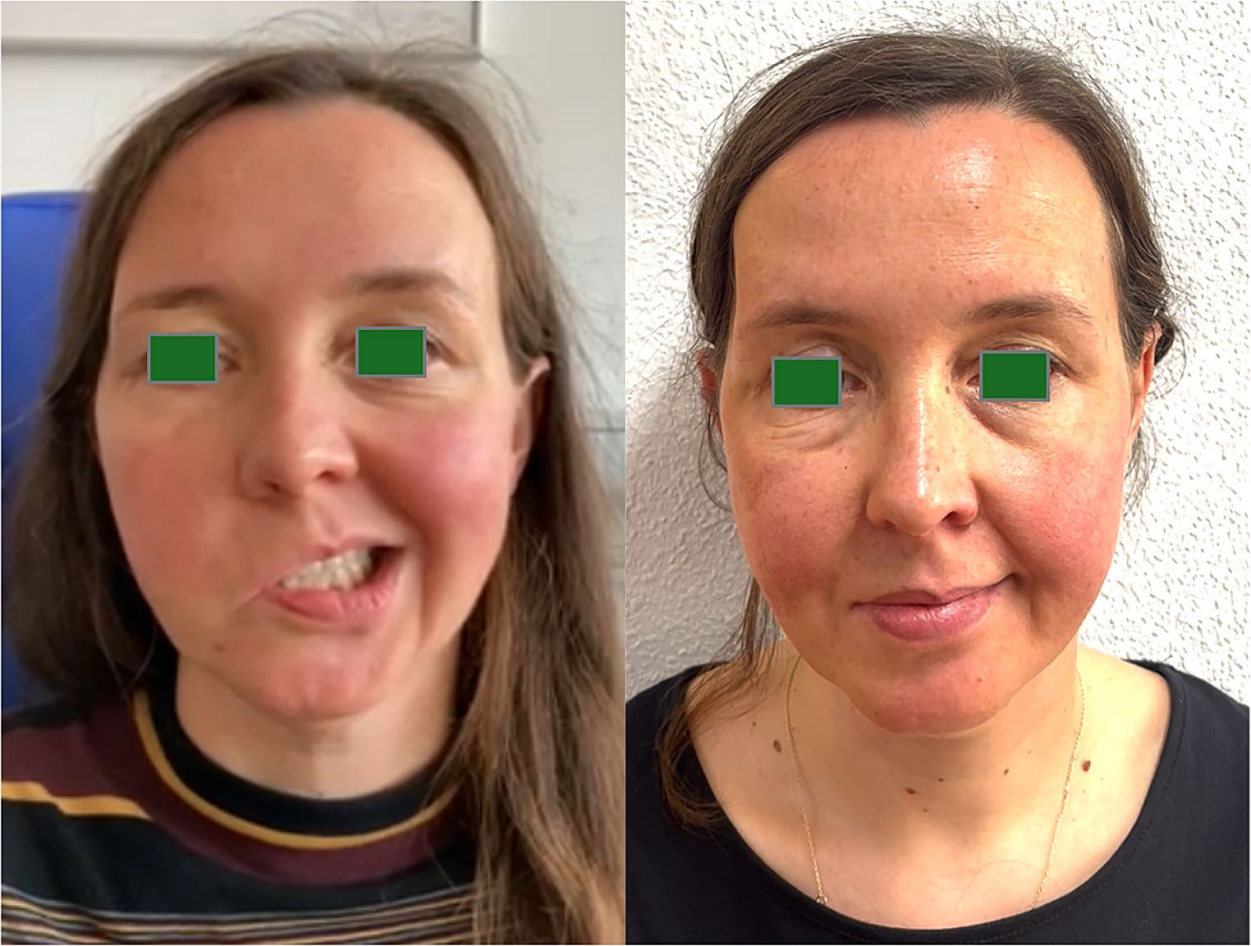
Patient; (left) before surgery with right-sided facial palsy, (right) after surgery 24 months after facial nerve reconstruction

We present the case of a 40-year-old female patient who visited our clinic complaining of slowly progressive paralysis of the right-sided facial muscles over the past 2.5 years, starting at the Mm. corrugator and orbicularis oculi, and progressing from the cranial to the caudal mimic muscles, finishing in complete facial palsy. The patient had no medical history and denied symptoms such as fever, night sweats or unintentional weight loss. Upon clinical examination, we observed a complete right-sided peripheral facial palsy (Fig. 1). All other otorhinolaryngological examination findings were within normal limits and blood tests showed unremarkable results.

**Fig. 2:**
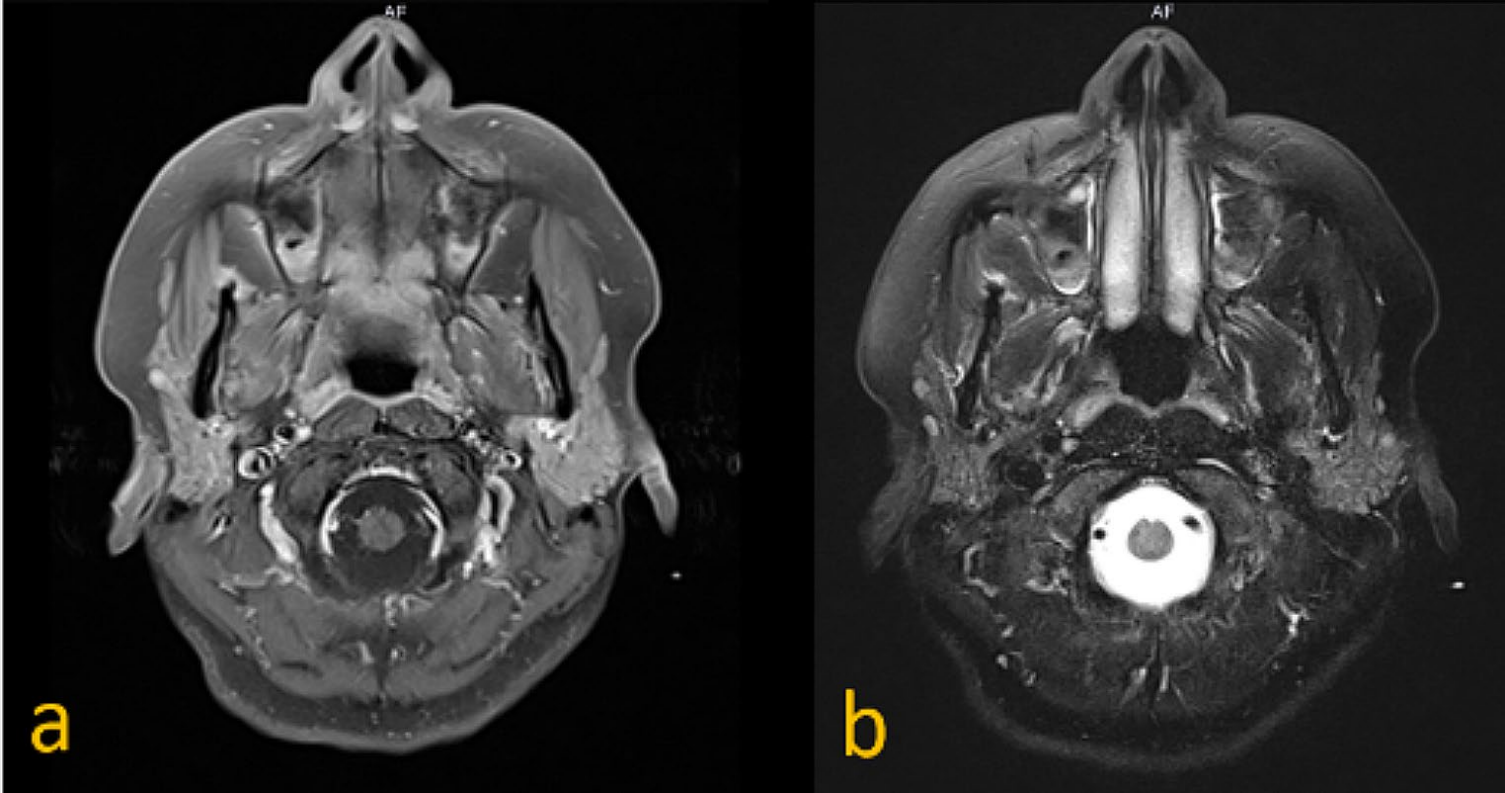
(**a**) MRI, T1, transversal; (**b**) MRI, T2, transversal. The right parotid gland shows even less parotid gland tissue then the left parotid gland. No lesions are depictable

**Fig. 3:**
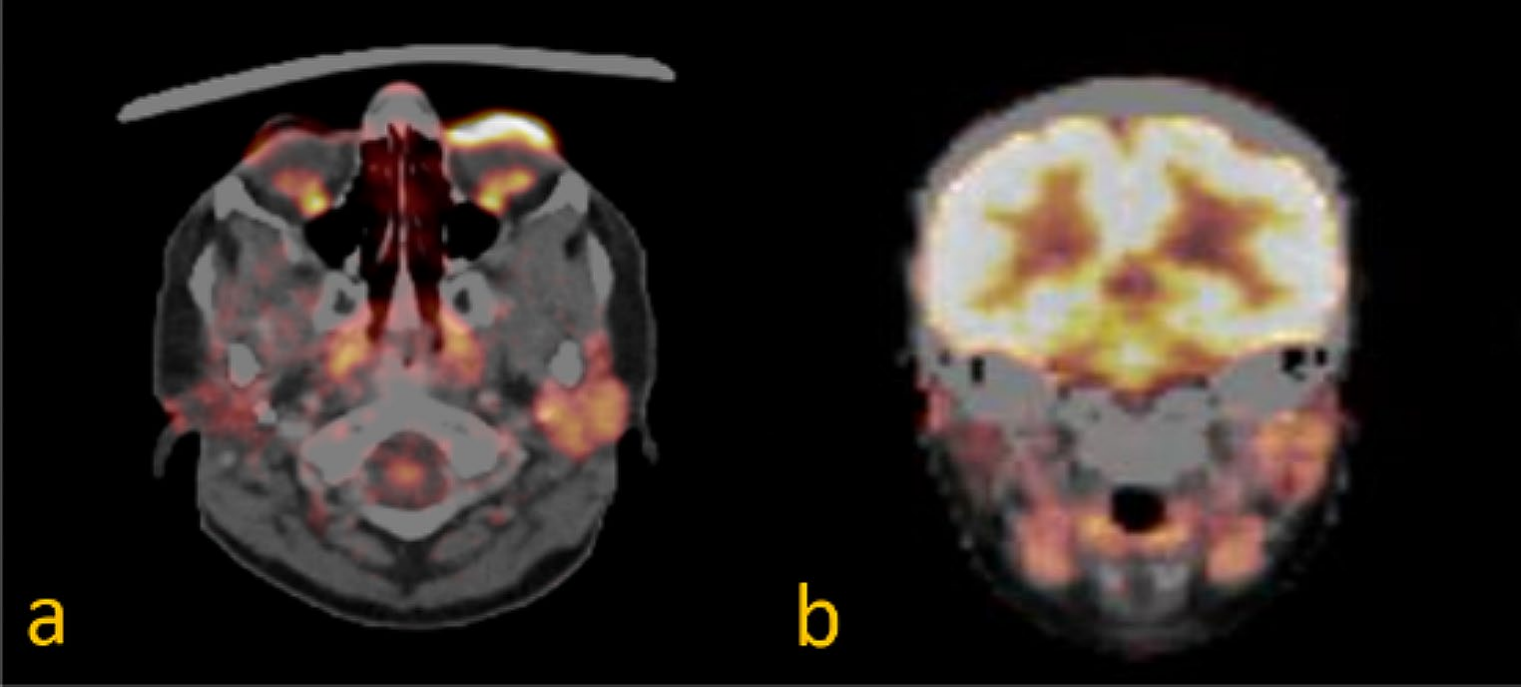
(**a**) 18-FDG PET-CT transversal; (**b**) 18-FDG PET-CT coronal; the right parotid gland shows even lower 18-FDG tracer uptake than the left side

Initially, magnetic resonance imaging (MRI) examination of the head and neck soft tissues (Fig. 2), computed tomography (CT) of the petrous bone and cranial base, as well as 18-fluorodeoxyglucose positron emission tomography-computed tomography (18-FDG PET-CT) (Fig. 3), were performed. They did not detect any cause for the peripheral facial palsy. There were no observable lesions, enlarged lymph nodes or pathological 18-FDG-uptake along the course of the peripheral facial nerve. The radiological examinations were evaluated by various highly experienced radiologists in the field of head and neck imaging at our certified head and neck tumor center.

**Fig. 4:**
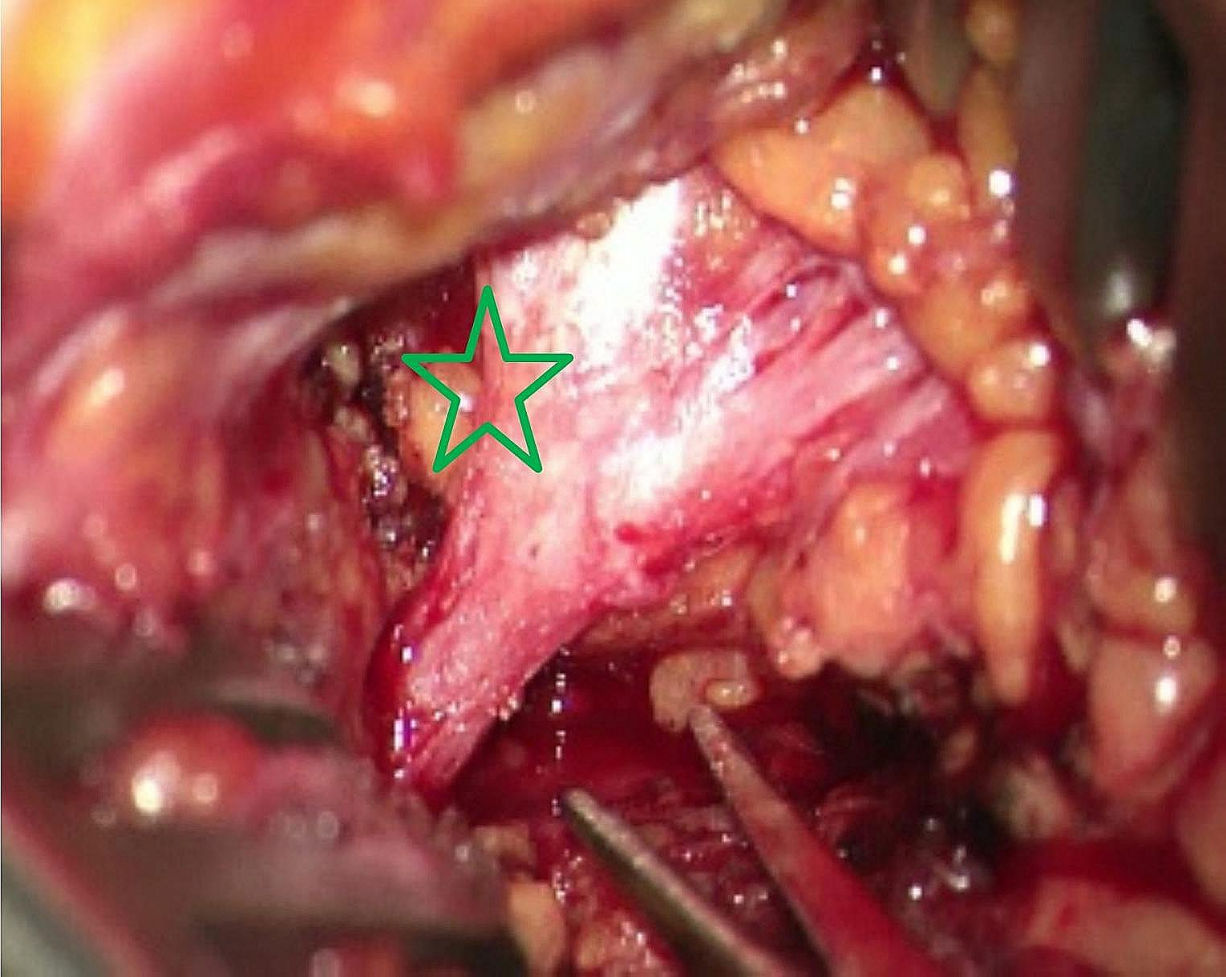
Main trunk of the facial nerve on the right in situ. Site of the biopsy (star). Biopsy was taken at the upper part of the bifurcation, as palsy started in the frontal mimic muscles

**Fig. 5:**
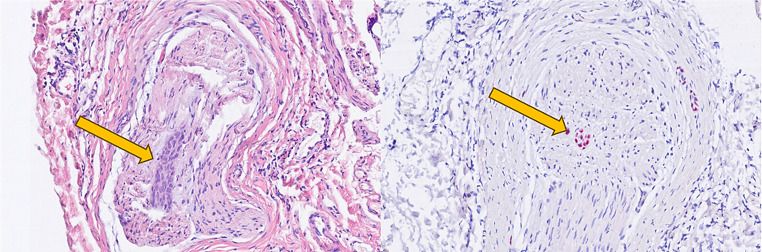
Hematoxylin-eosin staining, perineural and intraneural tumor infiltration (arrows); (left) 400x magnification, (right) 200x magnification

We decided to proceed with surgical exploration, which microscopically revealed no evidence of a tumor and an externally completely unremarkable facial nerve (Fig. 4). A biopsy from the main trunk area of the nerve close to its bifurcation revealed a perineural and intraneural infiltration by a p16-positive squamous cell carcinoma (Fig. 5). Following our interdisciplinary tumor board decision, a total parotidectomy with resection and reconstruction of the facial nerve using the diversification technique (auricularis magnus nerve interposition for the cranial facial nerve branches and hypoglossus-facial nerve jump anastomosis for the caudal facial nerve branches) and level II-III neck dissection were performed. After removal of the tumor-infiltrated main trunk, frozen sections were performed on the remaining nerve ends to ensure an R0 resection. Additionally, a platinum eyelid weight was implanted into the upper eyelid. The pathological examination revealed a moderately differentiated squamous cell carcinoma within the main trunk of the facial nerve without definite remnants of a primary tumor in the fully processed salivary gland (pT4a, pN0 (0/30), L0, V0, Pn1). Considering the absence of a primary tumor and other tumor formations in the otorhinolaryngological area, skin or other organs, the diagnosis of a completely regressive primary squamous cell carcinoma of the parotid gland was confirmed. Although adjuvant radiotherapy is usually performed in the case of malignant salivary gland tumor with perineural invasion, our interdisciplinary tumor board recommended a close follow-up care as an individual therapy plan due to the complete regression of the primary tumor and the sufficiently large safety margins. MRI of the soft tissues of the head and neck (after 3 months) and 18-FDG PET-CT (after 6 months) revealed no evidence of a primary tumor, locoregional lymph node involvement, distant metastases, or tumor recurrence. Whole-body CT (after 17 months) revealed no tumor regrowth or metastasis. Due to the immediate facial nerve reconstruction, the facial mimic recovered satisfactorily within 24 months, with complete eyelid closure and good facial tonus (Fig. 1).

## Discussion

In the case of slow-onset, progressive peripheral facial palsy, a gadolinium-enhanced MRI should be performed to exclude a malignant neoplasm. MRI is the method of choice especially for the evaluation of tumors of the stylomastoid foramen and the retromandibular part of the parotid gland. MRI enables the detection of even small lesions in the parotid gland as well as along the entire course of the facial nerve due to its high soft tissue resolution. However, magnetic resonance imaging has shown limitations in the evaluation of perineural tumor spread causing facial palsy [3]. Therefore, a high-quality MRI should always be performed at a center with extensive experience in parotid gland pathologies and must always be interpreted in conjunction with the clinical presentation of facial palsy. In addition, 18-FDG PET-CT is known as a highly sensitive functional imaging method for the detection of malignant diseases. In some cases, however, there is no detection of malignant lesions even after extensive morphological and functional imaging. To our knowledge, the case shown here is the first case of intraneural squamous cell carcinoma of the facial nerve that showed unremarkable findings on 18-FDG PET-CT. Boahene et al. reported 15 patients with unilateral peripheral facial palsy due to occult malignancy, who showed unremarkable clinical and radiological findings (MRI and CT). A diagnosis was confirmed in 13 of these patients through exploration and biopsy of the facial nerve, with two patients requiring a second biopsy to establish malignancy [4].

Furthermore, as in the case presented here, carcinoma could not be ruled out with certainty even if surgical exploration revealed a parotid gland without any signs of a tumor and an externally unremarkable facial nerve. Eggerstedt et al. reported a case of slow-onset peripheral facial palsy due to intraneural squamous cell carcinoma, which was confirmed only by final histology [5]. Additionally, it is possible for certain areas of the nerve to be skipped during the spread of carcinoma. Therefore, obtaining a sufficiently large biopsy and performing a repeated biopsy are recommended, if a malignancy is highly suspected [2].

## Conclusion

In summary, in the case of slow-onset peripheral facial palsy that persists without signs of recovery, a gadolinium-enhanced MRI should be performed. If imaging is unremarkable and there is no primary tumor detection along the course of the facial nerve, a surgical exploration with biopsy of the facial nerve is necessary. The case presented demonstrates that even with an externally unremarkable nerve, normal MRI and 18-FDG-PET-CT findings, peri- and intraneural tumor spread is possible. Resection of the parotid gland and the facial nerve is necessary when a biopsy proves malignant growth. Immediate facial nerve reconstruction allows favorable functional and esthetic recovery of the face.
